# Effectiveness and implementation challenges of mobile low-dose computed tomography units for early lung cancer detection: a systematic review

**DOI:** 10.3389/fpubh.2026.1884561

**Published:** 2026-07-31

**Authors:** Xiujing Lin, Yonglin Li, Fangfang Wang, Jialing Guo, Yujia Chen, Rachel Arbing, Wei-Ti Chen, Feifei Huang

**Affiliations:** 1 School of Nursing, Fujian Medical University, Fuzhou, Fujian, China; 2 Joe C. Wen School of Nursing, University of California Los Angeles, Los Angeles, CA, United States

**Keywords:** cancer screening, consolidated framework for implementation research, implementation effectiveness, low-dose computed tomography, lung neoplasms

## Abstract

**Introduction:**

A mobile low-dose computed tomography (LDCT) unit is an effective strategy for early lung cancer detection, particularly in high-risk populations affected by social and healthcare disparities. However, existing reviews are limited by only focusing on diagnostic outcomes. This study aimed to evaluate the characteristics, effectiveness, and key influencing factors of mobile LDCT-based unit interventions.

**Methods:**

A literature search was conducted from the inception of each bibliographic database to March 1, 2025. Two independent reviewers performed the study selection, quality assessment, and data extraction. Methodological quality was assessed via the Cochrane Risk of Bias 2 tool for randomized studies and the ROBINS-I tool for non-randomized studies. A qualitative synthesis was used to analyze intervention characteristics and effectiveness. Influencing factors were synthesized via the Consolidated Framework for Implementation Research (CFIR).

**Results:**

Eighteen studies were included. Suspected cases ranged 23–847 (detection 1.06–16.37%), with confirmed cases 2–107 (0.33–4.33%). Five studies demonstrated economic efficiency for the mobile LDCT-based unit, with early detection yielding favorable cost ratios (¥11,143–16,950 per case) and substantial savings over late-stage treatment. Financial analyses indicated positive returns (e.g., net present value = $1 M; internal rate of return = 34.6%). Screen-detected cases reduced treatment costs by 56% and reduced hospital stays by 70%. Influencing factors, categorized via the CFIR, included intervention characteristics (*n* = 2), outer setting (*n* = 4), inner setting (*n* = 4), and characteristics of individuals (*n* = 2) but excluded implementation process (*n* = 0).

**Discussion:**

This review shows mobile LDCT-based units have the potential benefit for early lung cancer detection and economic efficiency, particularly for underserved populations by reducing access barriers. Future research should identify implementation factors and develop practical frameworks to optimize program delivery and impact.

**Systematic review registration:**

https://www.crd.york.ac.uk/PROSPERO/view/CRD420251026810, Identifier CRD420251026810.

## 1 Introduction

Lung cancer is the most commonly diagnosed cancer and the leading cause of cancer-related death worldwide (1). Approximately 75% of patients are diagnosed at an advanced or metastatic stage, resulting in a dismal 5-year survival rate of 0–13% (2). In contrast, early-stage detection can yield a survival rate of up to 80% (3). Multiple international guidelines have shown that low-dose computed tomography (LDCT) screening for high-risk populations significantly improves early detection and reduces mortality (4, 5). LDCT can detect stage I lung cancer in 70–85% of cases, lower lung cancer mortality by more than 30%, increase the 5-year postoperative survival rate to 70–80%, and achieve a cost-effectiveness ratio above 80% (6).

Despite its proven benefits, the global uptake of LDCT screening remains low. In the U. S., screening rates among eligible individuals were below 5% between 2010 and 2015, and remained under 16.4% in 2022 (7, 8). In China, although “early detection and early treatment” programs were introduced in rural and urban areas between 2005 and 2012, participation rates remain below 35% (9). These low rates highlight persistent social and healthcare inequities—such as limited access to care, insurance barriers, transportation difficulties, and time constraints—which disproportionately impact underserved groups, including racial/ethnic minorities, rural populations, and socioeconomically disadvantaged individuals (10).

Mobile LDCT-based unit screening offers an innovative approach to lung cancer detection by providing services directly to underserved rural and socioeconomically disadvantaged communities (11). By utilizing mobile screening units—vans equipped with LDCT scanners and staffed by healthcare professionals—barriers such as distance and cost are reduced, improving access and participation (12). Programs in countries such as Australia, Japan, Kenya, the United Kingdom, and Brazil have shown promising outcomes, including earlier detection, reduced healthcare costs, and improved survival (13). However, the results are mixed. For example, a U. S. study reported low participation among certain high-risk groups, citing unclear implementation strategies and persistent access barriers (14). These inconsistencies highlight the need for rigorous, evidence-based evaluations to assess the true impact and sustainability of mobile LDCT-based unit programs.

To date, three reviews have examined mobile cancer screening programs, with only one focusing specifically on mobile LDCT-based units. These include reviews on mobile mammography among underserved women and mobile units for breast, cervical, and colon cancer detection (15, 16). The most relevant review is a recent scoping review on how mobile units can advance lung cancer screening (13). However, these reviews have limitations: they only included English-language studies from 2017–2023, restricting temporal and geographic scope; they focused mainly on diagnosis rates, neglecting outcomes such as cost-effectiveness and adverse events, and they did not analyze facilitators and barriers through an implementation science lens, which is crucial for guiding future program development.

Therefore, this review aims to synthesize existing evidence to identify key factors influencing the implementation of mobile LDCT-based interventions across diverse settings, guided by implementation science frameworks. Specifically, the Consolidated Framework for Implementation Research (CFIR) will be used to examine barriers and facilitators across five domains (17). Additionally, the review will evaluate the effectiveness of mobile LDCT-based unit interventions in increasing lung cancer screening uptake, assess their economic value, and summarize reported adverse events—providing an evidence-based foundation for broader adoption of mobile CT screening programs.

## 2 Method

A systematic review design was employed in this study. The research protocol was pre-registered in the International Prospective Register of Systematic Reviews (PROSPERO) under the registration ID CRD420251026810.

### 2.1 Eligibility criteria

To better reflect real-world implementation and conserve personnel and material resources, the study population comprised adults eligible for lung cancer screening on the basis of national or international guidelines (e.g., aged 50–75 years with a cumulative smoking history over 20 pack-years in China) or asymptomatic individuals at average or increased risk (e.g., family history or occupational exposure). Interventions focused on promoting early lung cancer detection through mobile LDCT-based units or exploring barriers and facilitators of mobile LDCT-based unit programs. Studies solely aimed at increasing screening uptake through standard recommendations (e.g., health education) were excluded. Both within-group (pre-post) and between-group comparisons were analyzed. The main outcomes of this study were the barriers and facilitators of the implementation of mobile LDCT-based unit programs based on the CFIR framework and the effectiveness of these programs, including the number and frequency of lung cancer diagnoses. Secondary outcomes included additional factors that may reflect the effectiveness of these programs, including participant numbers, lung cancer screening cases, adverse events (e.g., false positives, false negatives, over-diagnosis), and economic effectiveness.

The eligible study designs were randomized controlled trials (RCTs) and quasi-experimental studies published in English or Chinese. Gray literature, duplicates, conference abstracts, reviews, protocols, editorials, inaccessible full texts, studies focused solely on CT imaging performance, and those using mobile screening with X-ray devices were excluded.

### 2.2 Search strategy

A comprehensive search was conducted across twelve electronic databases: PubMed, EMBASE, CINAHL, Web of Science, the Cochrane Library, the World Health Organization (WHO), the Chinese National Knowledge Infrastructure (CNKI), the Wanfang Database, the China Science and Technology Journal Database, SinoMed, the National Health Commission of the People’s Republic of China, and the Chinese Medical Journal Database. Studies published up to March 1, 2025 were included. The search strategies were tailored to the indexing and syntax of each database, with the detailed PubMed strategy provided in Appendix 1. Additional eligible studies were identified by manually screening reference lists and through targeted searches in Google Scholar.

### 2.3 Study selection and quality assessment

Two reviewers independently screened titles and abstracts using predefined selection criteria to determine study eligibility. Full-text articles of potentially relevant studies were then retrieved and re-evaluated. Study quality was independently assessed by the same two reviewers via standardized tools: the Cochrane Risk-of-Bias 2 (RoB 2) tool for randomized controlled trials (18) and the ROBINS-I tool for quasi-experimental studies (see Appendixes 2, 3) (19). Any disagreements were resolved through discussion with a third senior researcher.

### 2.4 Data extraction and synthesis

The data extracted included author, country, year of publication, study design, participant and intervention characteristics, control group details, key findings (e.g., barriers and facilitators to implementing mobile LDCT-based unit programs, frequency of lung cancer diagnoses), user experience and feedback, and information on adverse events. Any discrepancies were resolved through discussion among the research team. Owing to substantial heterogeneity in clinical characteristics and outcome measures, a meta-analysis was not feasible. Instead, a narrative synthesis of the quantitative data was performed.

Regarding the facilitators and barriers of LDCT-based unit programs, the CFIR includes five domains: intervention, outer setting, inner setting, individuals, and implementation process. Adopting a deductive approach, we coded determinants as facilitators or barriers. These were then mapped to CFIR constructs and categorized into the five domains. Initial analysis was performed by one reviewer and reviewed by the team, with refinements made through discussion. All reviewers received comprehensive training on the CFIR, led by implementation research experts and experienced authors. Two reviewers independently completed the coding process and any disagreements were resolved by consensus or by a senior author if needed.

## 3 Results

### 3.1 Search selection

**Figure 1:**
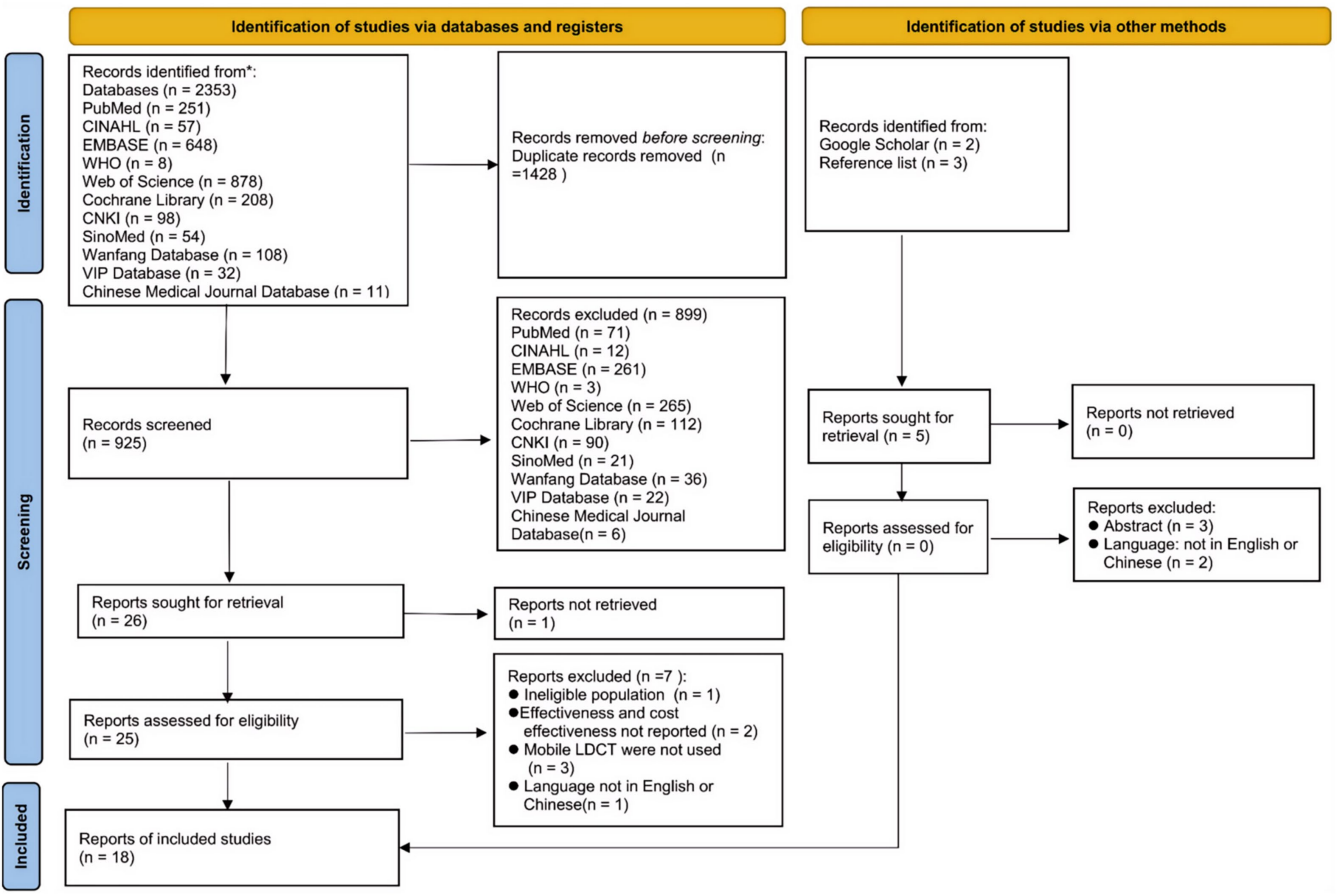
PRISMA flowchart for the systematic review. CINAHL, cumulative index to nursing and allied health literature; CNKI, Chinese national knowledge infrastructure; WHO, WHO global health library; VIP Database, China science and technology journal database.

The PRISMA flow diagram (Figure 1) summarizes the search process. A total of 2,358 records were identified, and after removing duplicates, 925 studies were screened. Following title, abstract, and full-text review, 25 studies met the inclusion criteria for quality assessment. Of these, 7 were excluded because of a high risk of bias. Ultimately, 18 studies were included: 1 randomized controlled trial (low risk of bias) and 17 quasi-experimental studies (low to moderate risk of bias).

### 3.2 Study characteristics

**Figure 2:**
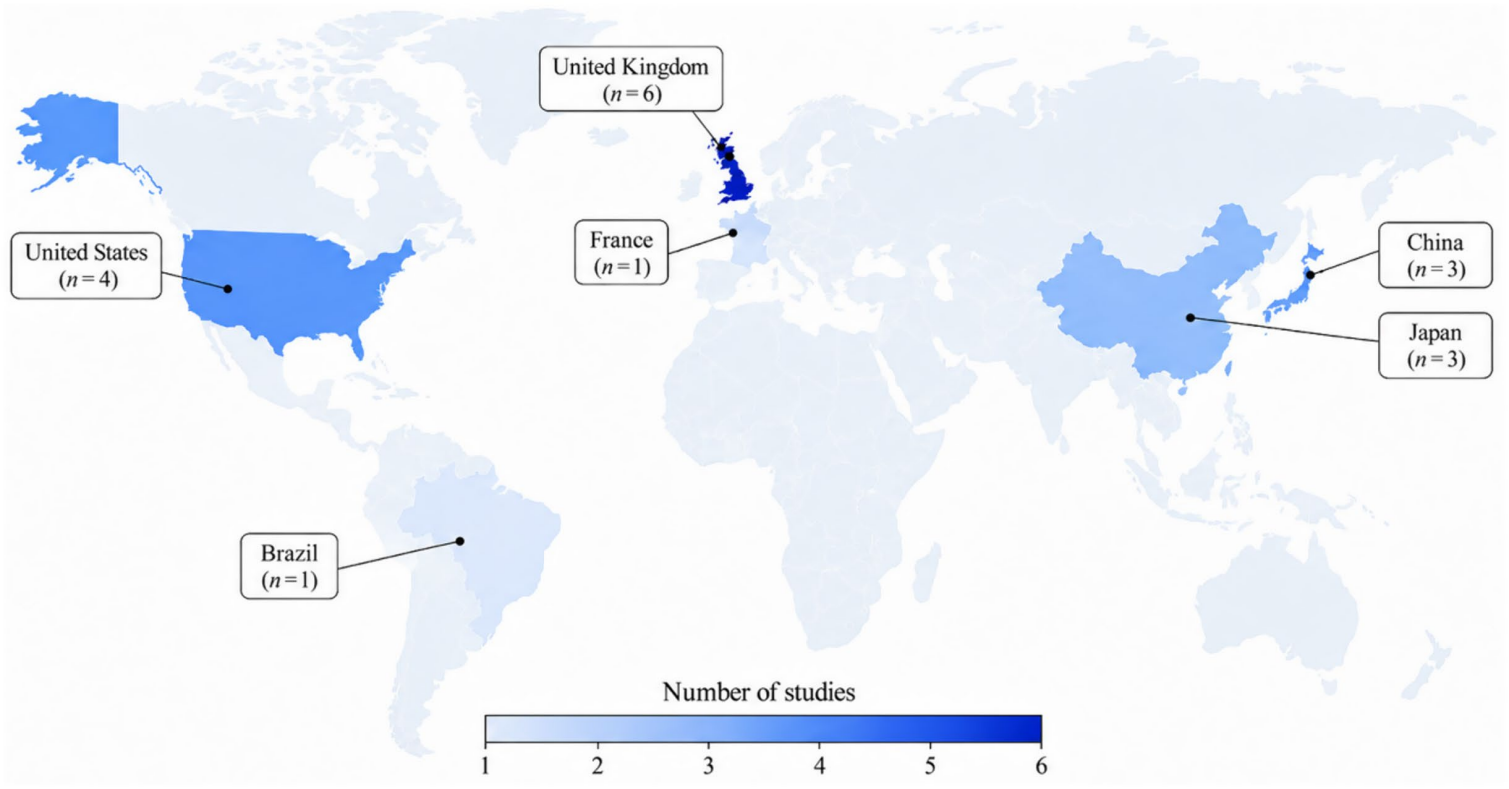
Countries conducting mobile LDCT unit programs.

The 18 included studies were published between 1998 and 2025 (Table 1) (14, 20–36) with 13 (72%) published in the last five years (14, 21–23, 27, 29, 31–36). One study (6%) was an RCT (29), whereas the remaining 17 (94%) were pre-post designs (14, 20–28, 30–36). Studies originated from six countries—Japan, China, the United States, the United Kingdom, Brazil, and France (Figure 2). Research sites were primarily in rural or deprived areas (*n* = 9, 50%) (20, 22–26, 28, 31, 36) followed by mixed rural–urban areas (*n* = 7, 39%) (21, 27, 29, 30, 32–34) and urban areas (*n* = 2, 11%) (14, 35). Study duration ranged from 2–120 months (14, 33), with over half (*n* = 9, 50%) lasting one to two years (24, 25, 27, 28, 31, 32, 34–36). The number of participants varied widely, from 216 to 25,189 (14, 33) with five studies (28%) enrolling over 10,000 subjects (21, 22, 30, 33, 36) and eight (44%) fewer than 1,000 (14, 23, 26, 27, 31, 32, 34, 35). Participants’ mean ages ranged from 58 to 68 years (22, 27). Most studies (*n* = 10, 56%) enrolled individuals meeting high-risk lung cancer criteria, defined by risk models (e.g., PLCOm2012, Liverpool Lung Project) or national guidelines (e.g., U. S. Preventive Services Task Force (USPSTF) 2013/2021, National Lung Screening Trial standards) (14, 24–29, 32, 34, 35). Other studies used simpler criteria such as age thresholds (≥40 or 50 years), smoking history (>30 pack-years), or voluntary participation (14, 21–23, 30, 31, 33, 36).

**Table 1:** Study characteristics (*N* = 18).

Study	Study design	Study implementation information				Characteristic of included sample				
		Study location	Study duration	Screening round	Screening interval	Eligibility	Sample size (attender/invitee)	Male (%)	Mean age (SD)	Proportion of high-risk participants (%)
Takizawa et al. (20)	Quasi-experimental	Rural (29 administrative districts)	11 mos	1	/	•Heavy smokers •50–85 years old	6358/21066	3,560 (56%)	NR	NR
Tao et al. (21)	Quasi-experimental	Rural and Urban (5 streets and 3 towns)	27 mos	1	/	•≥ 40 years old	19,517/28728	13,470 (69.02%)	NR	NR
Shao et al. (22)	Quasi-experimental	Rural (5 streets)	6 mos	1	/	•≥ 40 years old	12,360/NR	8,382 (67.82%)	NR	NR
Allen et al. (23)	Quasi-experimental	Rural (37 countries)	4 mos	1	/	•Local residents	725/NR	NR	NR	NR
Crosbie et al. (24)	Quasi-experimental	Deprived communities	15 mos	2	12 mos	•Ever smokers •55–74 years old •PLCOm_2012_ ≥ 1.51%	2541/3041	1,245 (48.70%)	56.2%	56.2%
Crosbie et al. (25)	Quasi-experimental	Deprived communities	15 mos	2	12 mos	•Ever smokers •55–74 years old •PLCOm_2012_ ≥ 1.51%	1194/1323	587 (49.16%)	NR	NR
Balata et al. (26)	Quasi-experimental	Deprived communities	NR	/	/	•Ever smokers •55–74 years old •PLCOm_2012_ ≥ 1.51%	938/NR	463 (49.36%)	65.6 (5.4)	NR
Bartlett et al. (27)	Quasi-experimental	West London	24 mos	2	24 mos	•Ever smokers •60–75 years old •PLCOm_2012_ ≥ 1.51% •LLPv2 ≥ 2.0%	H: (1,047/NR) M: (702/NR)	H: (492,47.0%) M: (303,43.2%)	H: (67 ± 63.71) M: (68 ± 64.72)	100%
Bradley et al. (28)	Quasi-experimental	Deprived communities	18 mos	2	12 mos	•Ever smokers •55–74 years old •PLCOm_2012_ ≥ 1.51%	1,409/NR	NR	NR	100%
Crosbie et al. (29)	RCT	Rural and Urban	27 mos	1	/	Meet any of the 3 conditions: •USPSTF 2013 criteria •PLCOm2012 ≥ 1.51% •LLPv2 ≥ 5.0%	7853/6819	NR	66.1 (7.2)	100%
Takizawa et al. (30)	Quasi-experimental	Rural and Urban	4 years	2	12 mos	•≥ 50 years old •Chronic smokers	19,117/NR	10,478 (54.8%)	NR	100%
Headrick et al. (31)	Quasi-experimental	Rural	12 mos	1	/	•55–80 years old, •≥ 30 pack-years •Current smoker or quit <15 years	548/NR	258 (47%)	62	100%
Chiarantano et al. (32)	Quasi-experimental	Rural and Urban	19 mos	2	12 mos	•age 55–74 years •≥ 30 pack-year •Current smokers, or former smokers quit <15 years	233/NR	105 (45.1%)	62	100%
Hamaguchi et al. (33)	Quasi-experimental	Rural and Urban	10 years	2	/	•55–74 years old	25,189/NR	13,686 (54.3%)	61	NR
Raghavan et al. (34)	Quasi-experimental	Rural and Urban	12 mos	1	/	•Age 55–74 years •≥ 30 pack-year •Current smokers, or former smokers quit <15 years •Without insurance or Medicaid	550/NR	NR	61	100%
Grenier et al. (35)	Quasi-experimental	Urban	12 mos	1	/	•50–80 years old •≥ 20 pack-years •Current smokers or former smokers quit <15 years	477/586	235 (49%)	60	100%
Pua et al. (14)	Quasi-experimental	Urban	2 mos	1	/	•Ever smoker •50–80 years old	M (216/NR) H (128/NR)	M:112 (51.9) H:70 (54.7)	M: 60.4 (6.8) H: 64.6 (5.9)	100%
Huang et al. (36)	Quasi-experimental	Rural	12 mos	1	/	•Socioeconomically marginalized •≥ 40 years old •LDCT-unscreened	10,159/NR	5,201 (51.2%)	59.2 (3.1)	100%

### 3.3 Intervention characteristics

Table 2 summarizes the characteristics and outcome measures of the interventions. Healthcare providers—such as physicians, nurses, and radiologists—were the most common intervention deliverers (*n* = 18, 100%) (14, 20–36), followed by CT technicians (*n* = 4, 22%), community health workers (*n* = 3, 17%) (21–23), drivers (*n* = 3, 17%) (21, 22, 31), dentists (*n* = 1, 6%) (32), and pharmacists (*n* = 1, 6%) (32). All studies utilized spiral CT scanners, while many also incorporated communication technologies (*n* = 7, 39%) such as computers, video conferencing systems, fax machines, and satellite communication platforms (14, 20, 30, 33, 35, 36). One study (17%) employed additional electronic tools, including image printers, smoking cessation education devices, nurse navigation systems, and decision aids (34). Emergency treatment equipment was described in two studies (11%) (20, 30). Diagnostic approaches included both AI and manual interpretation (*n* = 5) (14, 21–23, 36) and in two studies (11%), deep learning-based systems for lung nodule detection (21, 22). The follow-up frequency varied, with most studies conducting annual follow-up (*n* = 12, 67%) (21–29, 32, 34, 35).

**Table 2:** Intervention characteristics and economic effectiveness.

Study	Implementation resources				Effectiveness of mobile CT-based programs			
	Supplementary equipment	Team staff	Image reading method	Follow-up duration	Suspected lung cancer cases (n/total)	Lung cancer diagnosed cases (n/total)	Incidental finding (n)	Economic effectiveness finding
Takizawa et al. (20)	•Computer communication system •Video conference system •Fax machine •Emergency treatment set (oxygen inhaler, medicine, ECG) •On-line communication satellite	Radiologist	Manual review	/	226/6358	NR	•Tuberculosis (373) •Emphysema (279) •Atypical mycoplasma pneumonia (86) •Mid-lobe syndrome (226) •Bronchitis (690) •Pleural thickening (39) •Atelectasis (242) •Gall stones (40) •Liver cyst (175)	•Mobile hospital cost: 44% of conventional ($13,719 vs. $31,245) •Surgical hospital stay: 22.8 days (30% less vs. conventional 76.6 days)
Tao et al. (21)	•Two-stage deep learning model diagnosis system	Doctor Nurse Radiologist Driver CT technician Local coordinator	Manual+AI review	12 mos	524/19517	107/19517 0.55%	NR	NR
Shao et al. (22)	•Two-stage deep learning model diagnosis system	Doctor Nurse Radiologist Driver CT technician Local coordinator	Manual+AI review	12 mos	NR	86/8382 1.02%	NR	NR
Allen et al. (23)	/	Industry partner (provide mobile screen unit) Cancer institute Public primary care professional	Manual+AI review	7 mos	100/725 13.8%	6/725 0.83%	NR	NR
Crosbie et al. (24)	NR	Nurse Radiologist	Manual review	12 mos	176/2541 6.93%	42/2541 1.65%	NR	NR
Crosbie et al. (25)	NR	Nurse Radiologist	Manual review	12 mos	71/1194 5.95%	19/1194 1.59%	NR	NR
Balata et al. (26)	NR	Nurse Radiologist	Manual review	12 mos	NR	NR	NR	NR
Bartlett et al. (27)	NR	Radiologist	Manual review	24 mos	163	29	NR	NR
Bradley et al. (28)	NR	Nurse Radiologist	Manual review	18 mos	NR	NR	•Extrapulmonary malignancies (5) •Aortic dilation (19) •Lung consolidation (32) •Bronchiectasis (5)	NR
Crosbie et al. (29)	NR	Nurse Clinical trial assistant	NR	12 mos	NR	NR	Nr	NR
Takizawa et al. (30)	•Satellite communication systems •Video conference equipment •Fax machines •Resuscitation equipment •Personal computer •Video cameras •Image printer •Electronic lift •Lighting equipment •Rain guard	Driver	Manual review	NR	NR	75/19117 0.39%	Other lung/heart diseases found in 37% of screened	•Cost of each CT screening = $116 per person •Treatment cost: 45.1% of 1997 level •Treatment cost: 35.0% of 1998 level
Headrick et al. (31)	Data mining software	Driver CT technician Nurse Thoracic surgeon	Manual review	NR	54/548 9.85%	5/548 0.91%	Non-pulmonary significant abnormalities (e.g., coronary artery disease)(152)	•Downstream revenue per screened patient = 587 per screened patient •5 years net present value of $1 million
Chiarantano et al. (32)	NR	Doctor Nurse Dentist Pharmacist Public primary care professional	Manual review	Annually for high-risk individual	38/233 16.37%	3/233 1.29%	•Aortic dilation (19) •Emphysema (54.9%) •Coronary artery calcification (62.7%)	NR
Hamaguchi et al. (33)	NR	Pulmonologist	Manual review	NR	847/25189 3.36%	83/25189 0.33%	•Coronary artery calcification (62.7%) •Emphysema (54.9%) •Bronchial wall thickening (57.1%)	NR
Raghavan et al. (34)	•Electronic smoking cessation education tool •Electronic navigation system •Shared decision-making video aid	Pulmonologist Radiologist Oncologist	Manual review	12 mos	38	12/550 2.18%	•Coronary heart disease (16%) •Atherosclerosis (27%) •Pancreatic cancer (1)	•Early treatment costs = $300,000–$400,000, significantly less than late treatment (> $2 million)
Grenier et al. (35)	Standardized reporting system	Radiologist Pulmonologist Thoracic surgeon GP	Manual review	12 mos	23/477 4.82%	8/477 1.68%	•Emphysema (men 25.2%, women 12%) •Osteoporosis (men 13.2%, women 21.1%) •Coronary artery calcification (men 69.3%, women 58.1%)	NR
Pua et al. (14)	Standardized reporting system	Nurse CT technician navigator Radiologist	Manual+AI review	NR	25/216 11.57%	2/216 0.93%	•Coronary artery calcification (32) •Emphysema (9) •Pneumonia (17) •Abdominal abnormalities (14)	NR
Huang et al. (36)	Standardized reporting system	Doctor Nurse Radiologist Navigator	Manual+AI review	NR	108/10159 1.06%	71/10159 0.70%	NR	•Total cost = ¥1,203,504 •Per capita cost = ¥118.47 •EDCI (suspected) = 0.09 •EDCI (confirmed) = 0.13

### 3.4 The facilitators and barriers to mobile CT-based programs

**Figure 3:**
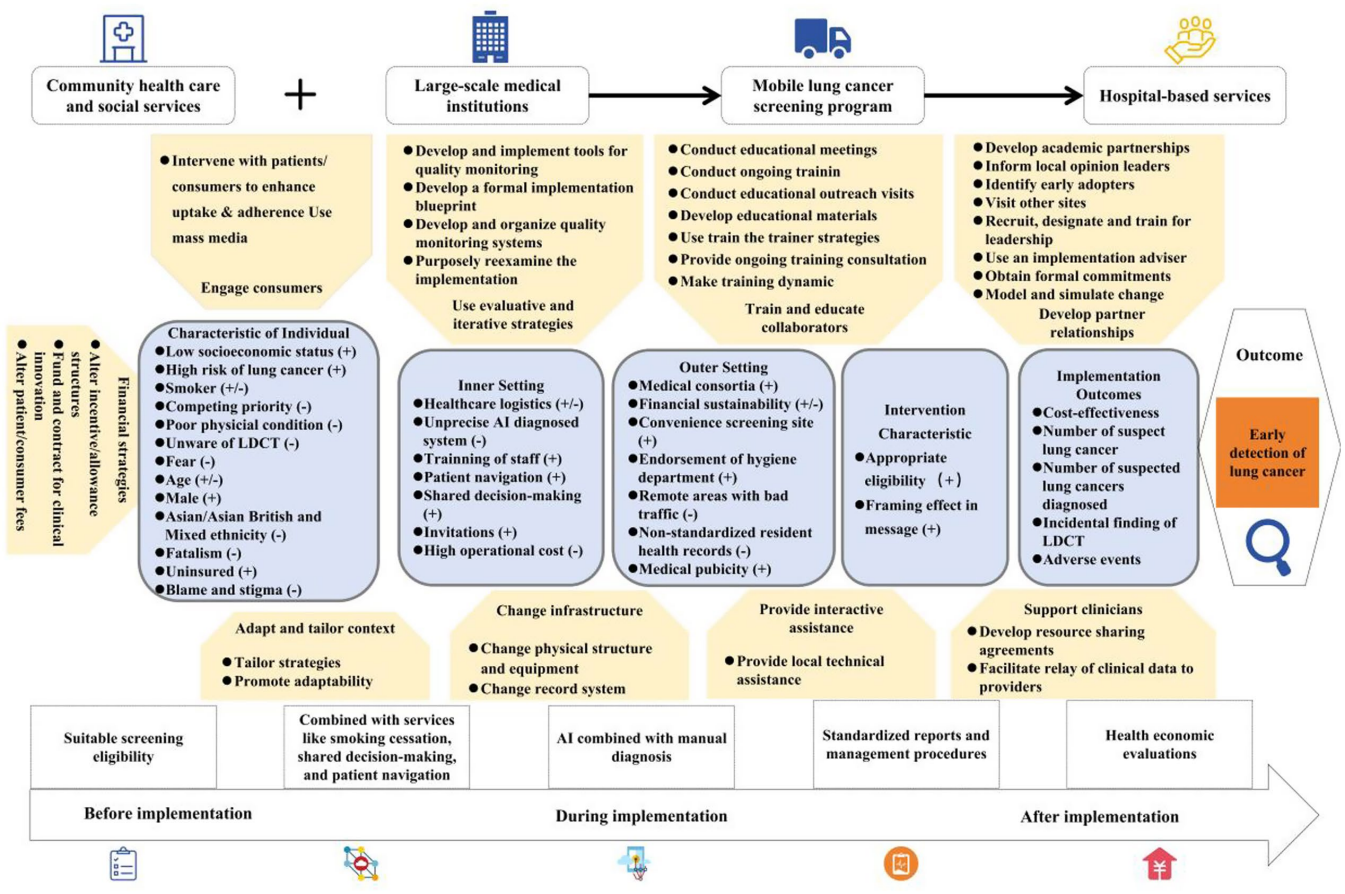
The influencing factors identified by the CFIR framework.

Factors influencing the implementation of mobile CT-based programs were categorized according to the CFIR. These factors were organized into five domains: intervention characteristics, inner setting, outer setting, characteristics of individuals, and the implementation process (Figure 3). The facilitators and barriers identified by each domain of the CFIR framework are summarized in Table 3. The detail of extracted facilitators and barriers from every included study are provided in Appendix 4.

**Table 3:** A summary of the facilitators and barriers as defined by the CFIR framework.

Domain	Facilitators	Barriers
Intervention characteristics	Broader eligibility criteria Framing effect (focused on benefits of LDCT)	Hard to make appropriate eligibility criteria
Outer setting	Telemedicine Hospital consortium collaboration Financial sustainability (philanthropic funds) Convenience screening site (local shopping centers, community locations, high-traffic and close proximity to public transportation station) Endorsement from hygiene department Media publicity	Poor financial sustainability (substantial infrastructure, recurring costs) Remote areas with bad traffic conditions Non-standardized management of health records
Inner setting	Staff training (identify eligible patients, shared decision-making discussion, referrals, patient registration, primary and secondary prevention knowledge, screening flow, LCS training and awareness sessions) Patient navigation Invitations (invitation letter, telephone triage invitation) Healthcare logistics (self-leveling, independent power, climate control, patient comfort, and drivability of vehicle) Reminder emails Shared decision-making (screening protocol, possibility of detecting abnormalities with LDCT, potential need for additional procedures) Multi-stakeholder team (nurse practitioner, navigator, radiologist, CT technologist, patient coordinator, call center worker)	Poor healthcare logistics (unstable internet connectivity, poor climate control for sensitive equipment, mobile unit is not continuously powered, transmission delay, mechanical issues of mobile vehicle, service area of the bus restricted to a 2-h radius) Imprecise AI diagnosed system High operational costs (equipment repairs, mobile LDCT renting) Insufficient human resources (doctors, nurses, radiologists, community healthcare providers, and community volunteers)
Characteristics of individuals	Low socioeconomic status High risk of lung cancer Smoker Older age Male Uninsured	Fear (claustrophobic, misunderstanding about a second scan, false-positive results) Competing priority (work commitment) Poor physical condition Smokers Ethnicity (Asian/Asian British and mixed compared with White) Fatalism Blame and stigma (afraid to admit to smoking status) Older age Unaware of LDCT (inclusion criteria, benefit)
Implementation process	-	-

#### 3.4.1 Intervention characteristics domain of CFIR

Within the intervention characteristics domain of the CFIR framework, two key constructs were identified as influencing factors. First, under complexity, message framing highlighted the benefits of lung cancer screening—such as their free and convenient access, the curability of early-stage lung cancer, and their life-saving potential—was reported as a facilitator (*n* = 1) (14). Second, the definition of high-risk groups (*n* = 3) emerged as a critical factor (14, 31, 32). For instance, one study applied the expanded 2021 USPSTF guidelines, which target individuals aged 50–80 years with a ≥ 20 pack-year smoking history, including those who had quit within the past 15 years. Compared with the 2013 criteria (age ≥ 55 years and ≥ 30 pack-years), broader eligibility improved inclusivity and enhanced access among underserved populations (14). Notably, seven other CFIR constructs in this domain—intervention source, evidence strength and quality, relative advantage, trialability, design quality and packaging, and cost—were not addressed in the included studies.

#### 3.4.2 Inner setting domain of CFIR

Within the available resources construct of the CFIR framework, studies have consistently emphasized the critical role of robust healthcare logistics in supporting mobile LDCT-based unit programs (21, 23, 30, 31, 36). A well-equipped mobile unit—featuring mobility, self-leveling capabilities, patient comfort, ease of drivability, and an independent power supply—was identified as a key facilitator. The training of staff was identified as a key facilitator (*n* = 4) (23, 30, 31, 36), covering program strategy, prevention roles, high-risk identification of eligible participants, patient registration, the role of primary and secondary prevention, screening workflows, shared decision-making, and referral pathways. Effective shared decision-making (*n* = 2) enables informed, value-based choices by clearly communicating screening benefits (e.g., early detection) and limitations (e.g., false positives, overdiagnosis) (14, 35). Patient navigation strategies (*n* = 3) enhanced care access, protocol adherence, and follow-up (14, 23, 34). For example, a United Kingdom study using practice-endorsed invitations and simplified leaflets reported a 7% increase in response rates. Additionally, invitations to potential participants were also identified as a facilitator (*n* = 2) (29, 35), including invitation letters, telephone triage invitations and emails.

In contrast, several healthcare logistical barriers have been reported, including unreliable internet connectivity (*n* = 2) (21, 30), inadequate climate control affecting sensitive equipment (*n* = 2) (21, 31), mechanical issues (*n* = 1) and operational constraints such as limiting the unit’s travel range to a two-hour radius (*n* = 1) (31), which impedes follow-up for those living farther away. Technological limitations emerged as a significant barrier. One study in China (*n* = 1) reported that an imprecise AI diagnostic system, unable to detect nodules smaller than 3 mm, led to a high rate of false positives, thereby compromising program feasibility (22). The high operational cost of mobile LDCT-based unit programs has also been reported as a barrier (*n* = 4) (30, 32, 35, 36).

#### 3.4.3 Outer setting domain of CFIR

For the CFIR patient needs and resources construct, convenience screening sites (such as retail locations, communities, stations and shopping centers) (*n* = 7) were the most frequently identified facilitators (14, 24, 25, 27, 29, 31, 32) with studies from the United Kingdom and the United States highlighting that convenient community sites can reduce travel barriers and improve service accessibility.

External policies and incentives also played a key role. Telemedicine and healthcare alliances (regional hospital networks) (*n* = 3) facilitated program implementation by enabling remote consultations with specialists and seamless referrals within regional hospital networks, particularly benefiting rural populations (21–23). One study partnered with a public screening division to promote CT screenings through municipal outreach and occupational health exams, encouraging eligible participants to apply. Financial sustainability was another common influencing factor, cited in three studies (21, 23, 31). For example, a study highlighted the substantial infrastructure and recurring costs associated with national mobile LDCT-based unit programs, underscoring the tension between resource allocation and the impact on public health (23). One study identified nonprofit funding for mobile screening vehicles as another facilitator (21). The management of resident health records (*n* = 1) was critical (36), as a lack of standardized records caused inconsistent participant selection, including the exclusion of some high-risk individuals. Media publicity (*n* = 5) was essential for program success (14, 29, 31, 35, 36). For example, a Chinese study reported that insufficient publicity undermined trust among high-risk groups, whereas other studies employed press, radio, TV, posters, and leaflets to attract more participants effectively. Additionally, studies have indicated that some areas are inaccessible to the mobile unit by having poor roads or bad traffic conditions (32).

#### 3.4.4 Characteristics of individuals domain of CFIR

Within the CFIR construct of knowledge and beliefs held by individuals involved in the intervention, four studies identified key barriers to implementing mobile LDCT-based unit programs. These included blame and stigma, fatalism, lack of awareness of LDCT (benefit, inclusion criteria, cost) among both patients and providers (14, 31), and fear (a second LDCT scan, false-positive result and claustrophobia) (14, 24, 26). For instance, one study found that many eligible individuals were never recommended for screening due to provider lack of awareness, despite proximity being a key facilitator (26). Another study noted that participation refusals due to claustrophobia (24).

Sociodemographic factors were identified as influencing factors in six studies, including medical insurance status (*n* = 2) (14, 34), smoking status (*n* = 3) (26, 27, 29), age (*n* = 3) (14, 27, 29), gender (*n* = 1) (29), race (*n* = 2) (14, 29), and social deprivation (*n* = 4) (14, 26–29). Males were more likely to attend screening than females were (*OR* = 1.33, 95% *CI*: 1.16–1.52) (26). Interestingly, uninsured individuals presented higher participation rates despite the availability of private insurance or Medicare. Compared to other racial groups, White individuals had higher screening rates. One study reported lower participation among Asian/Asian British and mixed ethnicity groups (*OR* = 0.79, 95% *CI*: 0.68–0.90) (14). Social deprivation was a strong motivator for mobile LDCT-based unit use—individuals in more deprived areas were more likely to use mobile LDCT-based units than hospital services, and those in the least deprived quartile were less willing to attend hospital-based screenings (*OR* = 2.0, 95% *CI*: 1.24–3.24) (24). Findings concerning age and smoking status were mixed. One study indicated that current smokers had significantly lower response rates (*OR* = 0.44, 95% *CI*: 0.42–0.46) (23) and another study indicated that fewer smokers preferred a hospital-based screening program (14). One study reported that participants in mobile LDCT-based unit programs were younger than hospital-based ones, whereas two other studies reported that older individuals preferred mobile sites (27). Additional barriers included competing priorities (*n* = 2) (14, 26), poor physical health (*n* = 1) (26) and high-risk of lung cancer (*n* = 1) (14).

#### 3.4.5 Implementation process domain of CFIR

In the CFIR process of implementation domain, five constructs—champions, formally appointed internal implementation leaders, planning, opinion leaders, and execution—were not addressed in the reviewed studies.

### 3.5 Effectiveness of mobile CT-based programs

As shown in Table 2, the number of suspected lung cancer cases across studies ranged from 23–847 (33, 35), with detection rates varying between 1.06 and 16.37%. The number of confirmed lung cancer cases ranged from 2 to 107 (14, 21), the rates ranging from 0.33% (33) to 2.18% (34).

### 3.6 The economic value and the incidental findings of mobile CT-based programs

Five studies evaluated the economic value of mobile LDCT-based unit screening (20, 30, 31, 34, 36). These outcomes fell into three categories: cost analysis, cost-effectiveness analysis, and cost–benefit analysis. No cost-utility analysis (e.g., cost per quality-adjusted life years) was identified.

Regarding to cost analysis, three studies reported cost or resource use outcomes without effectiveness denominators. Takizawa et al. reported that mobile screening reduced hospital stays by 26–28% and lowered per-patient costs to $9,896–$13,719 compared with $28,280–$30,394 in symptomatic patients (20). In later study, Takizawa et al. reported 56% lower treatment costs ($13,719 vs. $31,245) and 70% shorter postoperative stays (22.8 vs. 76.6 days) for screen-detected cases (30). Raghavan et al. noted that early-stage curative surgery ($300,000–$400,000) was significantly less costly than late-stage treatment (> $2,000,000) (34). With regard to cost-effectiveness analyses, one study conducted a formal cost-effectiveness analysis. Huang et al. reported cost-effectiveness ratios (CERs) of ¥11,143.56 ($1,753.29) per suspected case and ¥16,950.76 ($2,669.94) per confirmed case, with effective detection cost indices (EDCIs) of 0.09 and 0.13, respectively (36). With respect to the cost–benefit analysis, one study reported cost–benefit outcomes. Headrick et al. reported a net present value (NPV) of $1,001,932, an internal rate of return (IRR) of 34.6%, and a profitability index (PI) of 2.20, all indicating positive net economic returns (31).

Ten studies (56%) reported incidental findings in mobile LDCT-based unit programs (14, 20, 28, 30–35). The incidental findings included pulmonary disease (*n* = 8) (bronchiectasia, tuberculosis, emphysema, respiratory tract inflammation, pleural diseases, middle lobe syndrome, pulmonary atelectasis, pulmonary consolidation), abdominal diseases (*n* = 2) (abdominal abnormalities, gallstones, liver cysts), cardiovascular disease (*n* = 8) (coronary heart disease, aortosclerosis), extrapulmonary malignancies (*n* = 2) (renal cell carcinoma, adenocarcinoma of prostate, hepatocellular carcinoma), and other diseases (*n* = 2) (such as osteoporosis, renal cyst, thyroid nodule, hyperthyroidism, vocal cord polyp).

Adverse events were reported in two studies (24, 26), including false positives (34–48%), pneumothorax post-CT-guided biopsy (1.23%), surgery-related mortality within 90 days (2.17%), and unnecessary surgery for benign lesions (5.26%).

## 4 Discussion

This systematic review provides early insights into the potential effectiveness of mobile LDCT-based unit programs for lung cancer detection and identifies key implementation factors via the CFIR framework. Compared with traditional hospital-based screening, mobile LDCT-based unit programs remains in an exploratory phase, with implementation limited to a few countries and insufficiently reaching globally dispersed high-risk populations. It was noted that the lack of standardized inclusion criteria across programs reduces screening efficiency, increases resource use, and hampers comparability across studies. While evidence points to a potential role for mobile LDCT-based units in early detection, data on cost-effectiveness remains limited and requires further validation. Through the CFIR lens, key factors influencing implementation were identified across four domains: intervention characteristics, outer and inner settings, processes of implementation, and characteristics of the individuals involved. To enhance feasibility and impact, future mobile LDCT-based unit programs should be tailored to these contextual factors.

Our findings revealed significant variability in participant criteria across mobile LDCT-based unit programs. Some offered free screening to all adults to increase coverage, similar to the 2015 USPSTF expansion, which increased eligibility by 30.3%, especially among men (37). However, broad inclusion may reduce cost-effectiveness by increasing demands on resources (38) and exposing low-risk individuals to harms such as radiation, false positives, overdiagnosis, and financial burden from uncovered diagnostic costs (39). To address these challenges, researchers urge more evidence on the risk–benefit ratio of lung cancer screening across diverse populations to refine eligibility criteria (12). While many programs rely on age and smoking history, these factors may miss high-risk groups such as never-smokers—who account for 10–25% of cases (1)—and those with occupational exposures or indoor air pollution increasing their lifetime risk to ≥ 1.3% (40). This suggests that current mobile LDCT-based unit programs may overlook many high-risk individuals. Combining validated lung cancer risk prediction models with existing guidelines could better identify those with non-traditional risk factors, enhancing screening coverage and effectiveness (41).

Few studies have integrated mobile LDCT-based units with smoking cessation, shared decision-making, or patient navigation. Smoking cessation alongside screening significantly reduces lung cancer mortality, a 10% increase in quitting can reduce deaths by 14% (42). Mobile LDCT units create teachable moments by raising risk awareness and motivating quitting, especially when suspicious lesions are found (43, 44). Shared decision-making, where patients and providers discuss risks and preferences, is essential for informed screening. It improves satisfaction, reduces anxiety, and enhances understanding (45, 46), and is mandated by many guidelines before LDCT (4, 5, 47). Mobile LDCT alone does not reduce mortality; timely follow-up treatment is critical (48). Patient navigation, an evidence-based approach, addresses healthcare barriers, facilitates timely diagnostic evaluation and treatment—expediting biopsies, reducing delays, and increasing treatment initiation within 90 days (48, 49). As mobile LDCT-based unit programs expand globally, theses services should be integrated as vital components of patient-centered care to maximize screening effectiveness and improve patient outcomes.

In our review, only half of the included studies reported incidental findings from LDCT. Beyond lung cancer detection, LDCT can identify other clinically significant abnormalities (detection rate: 7.5%) (50, 51), including cardiovascular and pulmonary diseases, thyroid disorders, and upper abdominal tumors (52)—conditions common in older heavy smokers, who are also at high risk for tobacco-related comorbidities (50, 53). LDCT thus offers a valuable opportunity for early cardiovascular risk intervention and remains cost-effective despite follow-up costs (53). However, standardized reporting and management protocols are lacking, and regulatory frameworks remain underdeveloped (54). Future research should focus on developing clinical guidelines to optimize patient outcomes, resource use, and overall clinical benefit.

Accurate pulmonary nodule assessment is critical for lung cancer diagnosis, but variability in appearance and growth patterns limits radiographic precision (55, 56). Diagnosis still relies on expert interpretation, yet manual annotation is time-consuming and error-prone (57, 58). AI has been widely adopted for nodule detection, classification, and prognosis (59), but challenges remain. First, AI improves sensitivity at the cost of specificity, increasing false positives. Second, AI cannot explain etiologies, making it insufficient as a standalone tool and human interpretation remains essential. Third, limited and heterogeneous training datasets restrict AI generalizability across diverse settings (60). Therefore, guidelines recommend human-AI collaboration to boost diagnostic accuracy and efficiency (61–63). Future research should rigorously assess the added value of AI-clinician integration in lung cancer screening and diagnosis.

Health economic evaluations are essential for guiding health policy and investment decisions (64), but our review identified limitations in assessments of mobile LDCT-based screening programs. Cost-effectiveness analysis (CEA), the most common approach, focuses on cost relative to outcomes but often excludes quality of life, uses inconsistent outcome measurements, and lacks standardized thresholds, limiting comparability of studies (65, 66). Cost-utility analysis (CUA), which incorporates quality-adjusted life years to better capture benefits like early diagnosis and treatment (67), is less frequently used. We therefore recommend future mobile lung cancer screening (LCS) studies adopt CUA to assess both survival and quality-of-life benefits more comprehensively. The analytic perspective is also critical as it defines cost measurement scope and target audience (68). Societal, healthcare system, payer, and patient perspectives can shape economic evaluation differently. However, studies typically adopt only a single analytic perspective, limiting broader applicability. Future research should integrate multiple-perspectives in evaluations to better inform public health policy. Additionally, owing to the prolonged natural history of lung cancer, lengthy clinical studies are resource-intensive and often impractical. Modeling approaches, especially the Markov model, offer a practical alternative by simulating disease progression through stable transition probabilities between health states (69). This method enables accurate long-term cost-effectiveness estimates for mobile LDCT-based unit programs, overcoming traditional study limitations and supporting informed policy decisions.

Our review revealed that individual sociodemographic factors—such as age, gender, race, smoking status, insurance coverage, and socioeconomic status—were associated with mobile LDCT uptake, with some findings partially aligning with those of Lin et al. (10) but differing from those of Gebremeskel et al. (70) For example, unlike prior reviews showing higher LDCT participation among women, our results did not reflect this. Age findings were mixed: both younger and older participants preferred mobile sites, contrasting with studies showing no age-related differences in participation (71). While previous reviews reported lower adherence among current smokers, our review revealed that heavy smokers were more likely to engage with mobile LDCT. Socioeconomic status also diverged; prior studies cited financial hardship as a barrier, but we found greater deprivation was linked to greater mobile LDCT adherence. The race findings were consistent, with non-Whites being less adherent in both mobile and onsite programs. With respect to insurance, others reported coverage as a facilitator (72), yet uninsured individuals in our review favored free mobile LDCT. These differences highlight contrasts between onsite and mobile LDCT-based unit programs and suggest that future research should further explore how these demographic factors influence mobile LDCT uptake. Additionally, more modifiable psychological factors—such as lung cancer stigma, fatalism, lack of awareness, fear, concerns about false positives, and misunderstandings about follow-up—affect screening uptake and should be addressed in future interventions.

With respect to intervention characteristics, the gain-frame was identified as a facilitator, which is consistent with other reviews (73). The gain-frame effect, rooted in prospect theory, explains how people respond differently when health information is presented in terms of gains versus losses (74). Studies have shown that emphasizing benefits rather than risks can significantly influence patients’ treatment decisions, preventive behaviors, and adherence (75). Additionally, one study in our review revealed that more lenient inclusion criteria facilitated mobile LDCT participation, although this topic remains under-explored.

With regard to the inner setting, logistical support is fundamental for successful mobile LDCT implementation (76). Key factors include comprehensive transport maintenance (e.g., shockproof CT vehicles, backup generators), reliable internet, and climate control for sensitive equipment, all of which ensure operational continuity and increase daily screening capacity. Standardized logistical protocols are essential to maintain screening quality. Our findings also highlight that comprehensive training programs for healthcare providers significantly enhance mobile LDCT adoption. Provider education is a critical yet often overlooked component in early lung cancer detection (77), with persistent gaps in knowledge about screening effectiveness, risk–benefit communication, and eligibility (78). Programs based in the United States demonstrate that multimodal training—including protocol standardization and pre-service engagement—improves patient triage accuracy (79). This evidence supports the integration of structured educational modules to strengthen provider involvement in mobile LDCT services.

In terms of the outer setting, retail locations were identified as facilitators, consistent with two other reviews (10, 80). Retail sites increase LDCT participation among high-risk individuals through convenience, walk-in availability that reduces procrastination, lowers anxiety compared to clinical settings, and exerts social influence (81, 82). Telemedicine and healthcare alliances also support mobile LDCT-based programs by overcoming technological and geographic barriers (83) and through addressing resource allocation challenges (84), improving coverage and sustainability. Additionally, media publicity serves as a facilitator by disseminating health information, raising awareness, promoting behavior change (85), and reducing per-person recruitment costs (86). We recommend integrating multimedia strategies into mobile LDCT-based programs to engage more high-risk individuals.

Government funding is a key factor influencing mobile LDCT-based unit program implementation. Given persistently low screening uptake, especially in high-risk rural communities, integrating systematic LCS into national public health strategies is essential. Municipal authorities should allocate dedicated funding for mobile units providing integrated services to underserved areas. Our review also identified incomplete health records as a barrier to implementation. Electronic health records can effectively identify eligible individuals (87) highlighted the need for comprehensive healthcare data system restructuring across urban and rural settings. Regional providers should adopt standardized electronic health records with mandatory integration pathways to support screening and program coordination.

The implementation process domain of the CFIR plays a critical role in revealing pathways to success, driving continuous optimization, and ensuring effective implementation. However, no details related to the process domain have been identified in current mobile LDCT-based unit programs. This introduces uncertainty regarding the program’s successful replication and long-term sustainability.

This review has several limitations affecting generalizability. Limiting inclusion to English and Chinese studies could lead to the exclusion of relevant research in other languages. Second, the limited number of studies and available data precluded formal subgroup or stratified analyses, and substantial heterogeneity in baseline lung cancer risk across studies further reduced comparability. Third, this review primarily reports detection rates and case counts, which do not fully capture overall screening effectiveness. Comprehensive evaluation including data on stage distribution, diagnosis, treatment, mortality, long-term follow-up, and adverse events remains constrained. Fourth, the included studies span a long time period, during which CT technology, screening eligibility criteria, nodule management protocols, and healthcare delivery systems evolved substantially, introducing potential temporal confounding. Lastly, evidence on the economic valuation of mobile LDCT-based unit programs remains insufficient.

## 5 Conclusion

This review synthesizes findings from 18 studies to evaluate lung cancer screening programs that use mobile LDCT units. Key characteristics of the studies, the lung cancer screening effectiveness, and contextual factors affecting mobile LDCT unit program implementation were analyzed. The results indicate that mobile LDCT appears feasible and has strong potential to enhance early lung cancer detection, especially in underserved and rural populations, by addressing geographic and access barriers. However, current evidence remains heterogeneous and largely observational. Therefore, more robust evidence is needed to determine comparative effectiveness, harms, sustainability, and optimal implementation strategies. Future research should explore key determinants across five domains of CFIR and to promote a comprehensive implementation framework with practical recommendations to optimize mobile LDCT unit programs.

## Data Availability

The original contributions presented in the study are included in the article/supplementary material, further inquiries can be directed to the corresponding author/s.
