# Supplementary material for: Effectiveness and implementation challenges of mobile low-dose computed tomography units for early lung cancer detection: a systematic review
Source: Front Public Health. 2026 Jul 31;14:1884561. doi: 10.3389/fpubh.2026.1884561 (PMC13473411; doi:10.3389/fpubh.2026.1884561)
Supplement: Supplementary file 1 [file Table_1.docx]

**Appendix 1 Search strategy for PubMed**

| **#1** | “Mobile*” OR “Movable*” OR “Travel*” OR “Van*” OR “Vans” OR “Bus” OR “Buses” OR “Busses” OR “pre-hospital” OR “prehospital” [Title/Abstract] |
| --- | --- |
| **#2** | “Computed tomography” OR “Computerized tomography” OR “electron beam tomography” OR “tomodensitometry” OR “laminography” [Title/Abstract] |
| **#3** | “ct scan*” OR “cat scan*” OR “cine ct” OR “spiral CT” [Title/Abstract] |
| **#4** | “LDCT” OR “Low dose CT” OR “CT” [Title/Abstract] |
| **#5** | “spiral comput*” OR “helical comput* ” [Title/Abstract] |
| **#6** | "Tomography, X-Ray Computed"[Mesh] |
| **#7** | #2 OR #3 OR #4 OR #5 OR #6 |
| **#8** | "lung*" OR "pulmo*" OR "bronch*" [Title/Abstract] |
| **#9** | "cancer*" OR "neoplas*" OR "malignan*" OR "tumo*" OR "carcinoma*" OR "oncolo*" [Title/Abstract] |
| **#10** | #8 AND #9 |
| **#11** | "NSLC" OR "NSCLC" OR "SLC" OR "SCLC" [Title/Abstract] |
| **#12** | "Lung Neoplasms"[Mesh] |
| **#13** | #10 OR #11 OR #12 |
| **#14** | “screen*” OR “detect*” OR “diagnosis*” OR “prevent*” OR “test*” OR “discov*” OR “find*” [Title/Abstract] |
| **#15** | "Early Detection of Cancer"[Mesh] |
| **#16** | #14 OR #15 |
| **#17** | #13 AND #16 |
| **#18** | "Mobile Health Units"[Mesh] |
| **#19** | #1 AND #7 AND #17 |
| **#20** | #18 AND #17 |
| **#21** | #19 OR #20 |

**Appendix 2 Bias analysis results via version 2 of the Cochrane Risk-of-Bias tool for (RoB 2) for RCTs**

| **Bias domain** | **Signaling questions** | **Option** | **Crosbie et al.**  **2022** |
| --- | --- | --- | --- |
| Bias arising  from the  randomization  process | 1.1 Was the allocation sequence random? | Y/PY/PN/N/NI | Y |
|  | 1.2 Was the allocation sequence concealed until participants were recruited and assigned to interventions? | Y/PY/PN/N/NI | NI |
|  | 1.3 Were there baseline imbalances that suggest a problem with the randomization process? | Y/PY/PN/N/NI | N |
|  |  |  | **Low** |
| Bias due to  deviations  from intended  interventions | 2.1 Were participants aware of their assigned intervention during the trial? | Y/PY/PN/N/NI | N |
|  | 2.2 Were carers and trial personnel aware of participants' assigned intervention during the trial? | Y/PY/PN/N/NI | Y |
|  | 2.3 If Y/PY/NI to 2.1 or 2.2: Were there deviations from the intended intervention beyond what would be expected in usual practice? | NA/Y/PY/PN/N/NI | NI |
|  | 2.4. If Y/PY to 2.3: Were these deviations from intended intervention unbalanced between groups and likely to have affected the outcome? | NA/Y/PY/PN/N/NI |  |
|  | 2.5 If Y/PY/NI to 2.4: Did the deviation from established intervention measures occur equally across groups? | NA/Y/PY/PN/N/NI |  |
|  | 2.6 Was an appropriate analytical method used to estimate the effect of intervention measure allocation? | NA/Y/PY/PN/N/NI | Y |
|  | 2.7 If N/PN/NI to 2.6: Was there a potential for significant impact on the results due to misclassification of subjects during group analysis? | NA/Y/PY/PN/N/NI |  |
|  |  |  | **Some concern** |
| Bias due to deviating from established intervention measures (the effect of compliance with intervention measures) | 2.1 Were participants aware of their assigned intervention during the trial? | NA/Y/PY/PN/N/NI | Y |
|  | 2.2 Were carers and trial personnel aware of participants' assigned intervention during the trial? | NA/Y/PY/PN/N/NI | Y |
|  | 2.3 [If applicable] If Y/PY/NI to 2.1 or 2.2, were important unplanned interventions balanced between the groups? | NA/Y/PY/PN/N/NI | Y |
|  | 2.4 [If applicable] Could the non-completion of the intervention affect the outcome? | NA/Y/PY/PN/N/NI | N |
|  | 2.5 [If applicable] Could non-compliance with intervention measures potentially affect the outcomes of the participants? | NA/Y/PY/PN/N/NI | NA |
|  | 2.6 If N/PN/NT to 2.3. or Y/PY/NI to 2.4 or 2.5: Could non-compliance with intervention measures potentially affect the outcomes of the participants? | NA/Y/PY/PN/N/NI |  |
|  |  |  | **Low** |
| Bias due to  missing  outcome data | 3.1 Were outcome data available for all, or nearly all? | NA/Y/PY/PN/N/NI | Y |
|  | 3.2 If N/PN/NI to 3.1: Is there evidence that results were robust to the presence of missing outcome data? | NA/Y/PY/PN/N/NI |  |
|  | 3.3 If N/PN to 3.2: Is it possible for missing data to depend on its true value? | NA/Y/PY/PN/N/NI |  |
|  | 3.4 If Y/PY/NI to 3.3: Is the proportion of missing values in the outcome variable different between the two groups? | NA/Y/PY/PN/N/NI |  |
|  |  |  | **Low** |
| Bias in  measurement  of the  outcome | 4.1 Was the outcome measurement method inappropriate? | NA/Y/PY/PN/N/NI | N |
|  | 4.2 Was it possible for there to be inter-group differences in outcome measurement or determination? | NA/Y/PY/PN/N/NI | N |
|  | 4.3 If N/PN/NI to 4.1: Did the outcome assessors know which intervention measures the subjects have received? | NA/Y/PY/PN/N/NI | Y |
|  | 4.4 If Y/PY/NI to 4.3: Is it possible for the type of intervention received to affect the outcome measurement? | NA/Y/PY/PN/N/NI | PN |
|  | 4.5 If Y/PY/NI to 4.4: Did knowing the intervention measures affect the measurement of outcome variables? | NA/Y/PY/PN/N/NI |  |
|  |  |  | **Low** |
| Bias in  selection of  the reported  result | 5.1 Whether the data analysis of the results is consistent with the pre-determined analysis plan made before unblinding the outcome data? | NA/Y/PY/PN/N/NI | Y |
|  | 5.2 Multiple outcome measurements (e.g. scales, definitions, time points) within the outcome domain? | NA/Y/PY/PN/N/NI | N |
|  | 5.3 Multiple analyses of the data? | NA/Y/PY/PN/N/NI | N |
|  |  |  | **Low** |
| **Overall bias** |  |  | **Some concern** |

**Appendix 3 Bias analysis results via the Risk-of-Bias in Non-randomized Studies of Interventions (ROBINS-I) tool for quasi-experimental trials**

|  |  | **Question** | **Option** | **Takizawaet al.**  **1998** | **Tao**  **et al.**  **2023** | **Shao**  **et al.**  **2022** | **Allen**  **et al.**  **2022** | **Crosbie**  **et al.**  **2019** | **Crosbie et al.**  **2019** | **Balata**  **et al.**  **2019** | **Emily**  **et al.**  **2020** | **Bartlettet al.**  **2022** | **Masaomi**  **et al.**  **2001** | **Headricket al.**  **2020** | **Chiarantano**  **et al.**  **2022** | **Hamaguchi**  **et al.**  **2022** | **Raghavan**  **et al.**  **2020** | **Grenier**  **et al.**  **2024** | **Pua**  **et al.**  **2024** | **Huang**  **et al.**  **2025** |
| --- | --- | --- | --- | --- | --- | --- | --- | --- | --- | --- | --- | --- | --- | --- | --- | --- | --- | --- | --- | --- |
| 1. Bias due to confounding |  | 1.1 Were there potential confounding factors that may affect the intervention effect in the study? |  | N | N | N | N | N | N | N | N | N | N | N | N | N | N | N | N | N |
|  |  | 1.2 Was the outcome analysis based on the follow-up time periods that were split due to intervention conversion among the participants? | N/PN：respond  1.4-1.6  Y/PY：respond 1.3 |  |  |  |  |  |  |  |  |  |  |  |  |  |  |  |  |  |
|  |  | 1.3 Was it possible that the interruption or conversion of the intervention is related to the prognostic factors of the outcome? | N/PN：respond 1.4-1.6  Y/PY：respond 1.7-1.8 |  |  |  |  |  |  |  |  |  |  |  |  |  |  |  |  |  |
|  | Confounding only exists at baseline. | 1.4 Did the authors use appropriate analytical methods to control for all important confounding factors? | Y/PY：respond 1.5 |  |  |  |  |  |  |  |  |  |  |  |  |  |  |  |  |  |
|  |  | 1.5 Did the authors measure variables that are effective and reliable in controlling confounding variables in the study? |  |  |  |  |  |  |  |  |  |  |  |  |  |  |  |  |  |  |
|  |  | 1.6 Did the authors control for any post-intervention variables that may have been affected by the intervention? |  |  |  |  |  |  |  |  |  |  |  |  |  |  |  |  |  |  |
|  | Baseline confounding and time-varying confounding coexist. | 1.7 Did the authors use appropriate analytical methods to control for all important confounding variables and time-varying confounding? | Y/PY：respond 1.8 |  |  |  |  |  |  |  |  |  |  |  |  |  |  |  |  |  |
|  |  | 1.8 Did the authors effectively and reliably control for confounding variables by measuring variables that are available in the study? |  |  |  |  |  |  |  |  |  |  |  |  |  |  |  |  |  |  |
|  |  |  |  | **Low** | **Low** | **Low** | **Low** | **Low** | **Low** | **Low** | **Low** | **Low** | **Low** | **Low** | **Low** | **Low** | **Low** | **Low** | **Low** | **Low** |
| 2. Bias in selection of participants into the study |  | 2.1 Were the study participants selected for inclusion in the study or analysis based on individual characteristics observed after the intervention began? | N/PN: respond 2.4  Y/PY: respond 2.2 | N | N | N | N | N | N | N | N | N | N | N | N | N | N | N | N | N |
|  |  | 2.2 Were the intervention-related variables that influence the selection of participants potentially related to the intervention itself? | Y/PY: respond 2.3 |  |  |  |  |  |  |  |  |  |  |  |  |  |  |  |  |  |
|  |  | 2.3 Did the intervention-related variables that affect selection also potentially influenced by the occurrence or cause of the outcomes? | Y/PY: respond 2.5 |  |  |  |  |  |  |  |  |  |  |  |  |  |  |  |  |  |
|  |  | 2.4 Were most of the follow-up times for study participants consistent with the start time of the intervention? | N/PN: respond 2.5 | Y | Y | Y | Y | Y | Y | Y | Y | Y | Y | Y | Y | Y | Y | Y | Y | Y |
|  |  | 2.5 Could the correction techniques be used to correct for selection bias? |  |  |  |  |  |  |  |  |  |  |  |  |  |  |  |  |  |  |
|  |  |  |  | **Low** | **Low** | **Low** | **Low** | **Low** | **Low** | **Low** | **Low** | **Low** | **Low** | **Low** | **Low** | **Low** | **Low** | **Low** | **Low** | **Low** |
| 3. Bias in classification of interventions |  | 3.1 Was the intervention group clearly defined? |  | Y | Y | Y | Y | Y | Y | Y | Y | Y | Y | Y | Y | Y | Y | Y | Y | Y |
|  |  | 3.2 Was information on the definition of the intervention group recorded at the start of the intervention? |  | Y | Y | Y | Y | Y | Y | Y | Y | Y | Y | Y | Y | Y | Y | Y | Y | Y |
|  |  | 3.3 Did the understanding of the outcome or outcome risk affect the classification of the intervention? |  | N | N | N | N | N | N | N | N | N | N | N | N | N | N | N | N | N |
|  |  |  |  | **Low** | **Low** | **Low** | **Low** | **Low** | **Low** | **Low** | **Low** | **Low** | **Low** | **Low** | **Low** | **Low** | **Low** | **Low** | **Low** | **Low** |
| 4. Bias due to deviations from intended interventions | If the research objective is to evaluate the results of intention-to-treat analysis, refer to section 4.1; if the research objective is to evaluate compliance and analytical results, refer to section 4.3. | 4.1 Were there any deviations from expected practices in the intention of the intervention beyond routine clinical practice? | Y/PY: respond 4.2 | Y | Y | Y | Y | Y | Y | Y | Y | Y | Y | Y | Y | Y | Y | Y | Y | Y |
|  |  | 4.2 Were these deviations from intention interventions unevenly distributed among groups and may have affected the outcomes? |  |  |  |  |  |  |  |  |  |  |  |  |  |  |  |  |  |  |
|  |  | 4.3 Were the important accompanying interventions balanced among the intervention groups? | N/PN: respond 4.6 | Y | Y | Y | Y | Y | Y | Y | Y | Y | Y | Y | Y | Y | Y | Y | Y | Y |
|  |  | 4.4 Were the interventions successfully implemented for most of the participants? | N/PN: respond 4.6 | Y | Y | Y | Y | Y | Y | Y | Y | Y | Y | Y | Y | Y | Y | Y | Y | Y |
|  |  | 4.5 Did the study participants comply with the assigned intervention? | N/PN: respond 4.6 | Y | Y | Y | Y | Y | Y | Y | Y | Y | Y | Y | Y | Y | Y | Y | Y | Y |
|  |  | 4.6 Was appropriate analysis used in the study to assess the adherence effect of the intervention? |  |  |  |  |  |  |  |  |  |  |  |  |  |  |  |  |  |  |
|  |  |  |  | **Low** | **Low** | **Low** | **Low** | **Low** | **Low** | **Low** | **Low** | **Low** | **Low** | **Low** | **Low** | **Low** | **Low** | **Low** | **Low** | **Low** |
| 5. Bias due to missing data |  | 5.1 Was it possible to obtain outcome data for all or almost all participants in the study? | N/PN; respond 5.4-5.5 | Y | Y | Y | Y | Y | Y | Y | Y | Y | Y | Y | Y | Y | Y | Y | Y | Y |
|  |  | 5.2 Were participants excluded due to missing data on intervention status? | Y/PY: respond 5.4-5.5 | N | N | N | N | N | N | N | N | N | N | N | N | N | N | N | N | N |
|  |  | 5.3 Were participants excluded due to missing data on other variables required for analysis? | Y/PY: respond 5.4-5.5 | N | N | N | N | Y | N | N | N | N | N | N | N | N | N | N | N | N |
|  |  | 5.4 Was the proportion of missing data and the reasons for missing data similar between groups? |  |  |  |  | NI | N |  |  |  |  |  |  |  |  |  |  |  |  |
|  |  | 5.5 Was there evidence that the results remain robust after missing data? |  |  |  |  | N | N |  |  |  |  |  |  |  |  |  |  |  |  |
|  |  |  |  | **Low** | **Low** | **Low** | **High** | **High** | **Low** | **Low** | **Low** | **Low** | **Low** | **Low** | **Low** | **Low** | **Low** | **Low** | **Low** | **Low** |
| 6. Bias in measurement of outcomes |  | 6.1 Did knowledge of the intervention affect the measurement of the outcome? |  | NI | NI | NI | NI | NI | NI | NI | NI | NI | NI | NI | NI | NI | NI | NI | NI | NI |
|  |  | 6.2 Did outcome assessors know the interventions received by the participants? |  | N | Y | Y | Y | NI | N | N | N | N | N | N | N | N | NI | N | N | N |
|  |  | 6.3 Were outcome assessment methods comparable between intervention groups? |  | Y | Y | Y | Y | Y | Y | Y | Y | Y | Y | Y | Y | Y | Y | Y | Y | Y |
|  |  | 6.4 Was there any systematic error in outcome measurement related to the intervention received? |  | N | NI | NI | NI | N | NI | N | N | N | N | N | N | N | N | N | N | N |
|  |  |  |  | **Low** | **High** | **High** | **High** | **Low** | **Low** | **Low** | **Low** | **Low** | **Low** | **Low** | **Low** | **Low** | **Low** | **Low** | **Low** | **Low** |
| 7. Bias in selection of the reported result |  | 7.1 Was there any selective reporting of multiple outcome measurements within a specific outcome domain? |  | N | N | N | N | N | N | N | N | N | N | N | N | N | N | N | N | N |
|  |  | 7.2 Was there any selective reporting of effect sizes for multiple analyses of intervention-outcome relationships? |  | N | N | N | N | N | N | N | N | N | N | N | N | N | N | N | N | N |
|  |  | 7.3 Was there any selective reporting of different subgroups? |  | Y | Y | Y | Y | Y | Y | Y | Y | Y | Y | Y | Y | Y | Y | Y | Y | Y |
|  |  |  |  | **Low** | **Low** | **Low** | **Low** | **Low** | **Low** | **Low** | **Low** | **Low** | **Low** | **Low** | **Low** | **Low** | **Low** | **Low** | **Low** | **Low** |
| **Overall bias** |  |  |  | **Low** | **Low** | **Low** | **High** | **High** | **Low** | **Low** | **Low** | **Low** | **Low** | **Low** | **Low** | **Low** | **Low** | **Low** | **Low** | **Low** |

**Appendix 4 A summary of the facilitators and barriers as defined by the CFIR framework**

| Study | INTERVENTION CHARACTERISTICS | OUTER SETTING | INNER SETTING | CHARACTERISTICS OF INDIVIDUALS | PROCESS |
| --- | --- | --- | --- | --- | --- |
| Takizawa et al^20^  (1998, Japan) | - | - | - | - | - |
| Tao et al^21^  (2023, China) | - | **Facilitators**  ·Telemedicine  · Collaboration between top hospitals and local hospitals in medical consortia  **Barriers**  ·Financial sustainability | **Barriers**  ·Healthcare logistics (internet connectivity, climate control for sensitive equipment) | - | - |
| Shao et al^22^  (2022, China) | - | **Facilitators**  ·Telemedicine | **Barriers**  ·Unprecise AI diagnosed system | - | - |
| Allen et al^23^  (2022, United States) | - | **Facilitators**  ·Telemedicine  ·Financial sustainability | **Facilitators**  ·Staff training (identify  eligible patients, shared decision-making  discussions, and referrals)  ·Patient navigation  **Barriers**  ·Healthcare logistics (continuously powered  mobile unit) | - | - |
| Crosbie et al^24^  (2019, United Kingdom) | - | **Facilitators**  ·Convenience screening site (local shopping  centers) | - | **Facilitators**  ·Low socioeconomic status  **Barriers**  ·Fear (claustrophobic) | - |
| Crosbie et al^25^  (2019, United Kingdom) | - | - | - | - | - |
| Balata et al^26^  (2019, United Kingdom) | - | **Facilitators**  ·Convenience screening site (local shopping  centers) | - | **Facilitators**  ·High risk (PLCOm2012 score)  ·Smoker  ·Low socioeconomic status  **Barriers**  ·Competing priority (work commitment)  ·Poor physical condition  ·Fear (misunderstanding about a second scan) | - |
| Bartlett et al^27^  (2020, United Kingdom) | - | **Facilitators**  ·Convenience screening site (local shopping  centers) | - | **Facilitators**  ·Older age  ·Low socioeconomic status  **Barriers**  ·Smokers | - |
| Bradley et al^28^  (2022, United Kingdom) | - | - | - | - | - |
| Crosbie et al^29^  (2022, United Kingdom) | - | **Facilitators**  ·Convenience screening site (community  locations)  ·Endorsement from hygiene department (UK Health Research Authority)  ·Media publicity | **Facilitators**  ·Invitations (invitation letter, telephone triage invitation) | **Facilitators**  ·Low socioeconomic status  ·Older age  ·Male  **Barriers**  ·Asian/Asian British and  mixed ethnicity (compared with  White)  ·Smoker | - |
| Takizawa et al^30^  (2001, Japan) |  |  | **Barriers**  ·High operational costs (e.g., equipment repairs)  ·Healthcare logistics (transmission delay, unreliable internet connectivity) |  | - |
| Headrick et al^31^  (2020, United States) | **Barriers**  ·Hard to making appropriate eligibility criteria | **Facilitators**  ·Convenience screening site (local shopping  centers)  ·Financial sustainability (philanthropic  funds)  ·Media publicity | **Facilitators**  ·Healthcare logistics (self-leveling, independent power, climate control, patient comfort, and drivability vehicle)  ·Training to staff (drivers received patient registration training)  **Barriers**  ·Healthcare logistics (mechanical issues of mobile vehicle, service area of the bus  restricted to a 2-hour radius) | **Barriers**  ·Fatalism  ·Unaware of LDCT (benefits of LDCT  ·Blame and stigma (afraid to admit to smoking status) | - |
| Chiarantano et al^32^  （2022, Brazil） | **Barriers**  ·Hard to making appropriate eligibility criteria | **Barriers**  ·Remote areas with bad traffic conditions | **Facilitators**  ·Training to staff (healthcare provider receive  training on LCS approach, role of primary and secondary prevention, high-risk groups, and screening flow)  **Barriers**  ·High operational costs (mobile  LDCT) | - | - |
| Hamaguchi et al^33^  （2022, Japan） | - | - | - | - | - |
| Raghavan et al^34^  (2020, United States) | - | - | **Facilitators**  ·Patient navigation | **Facilitators**  ·Uninsured | - |
| Grenier et al^35^  (2024, France) | - | **Facilitators**  ·Public media | **Facilitators**  ·Staff training (LCS training and awareness sessions)  ·Explanations given to participants (screening protocol, possibility of detecting abnormalities with LDCT, and potential need for additional procedures)  ·Reminder emails  ·Shared decision-making  **Barriers**  ·High operational costs (mobile LDCT) | - | - |
| Pua et al^14^  (2024, United States) | **Facilitators**  ·Broader eligibility criteria  ·Framing effect (focused on benefits of LDCT) | **Facilitators**  ·Convenience screening site (high-traffic and close proximity to public transportation station)  ·Public media | **Facilitators**  ·Patient navigation  ·Shared decision-making  ·Multi-stakeholder team (nurse practitioner, nurse navigator, reading radiologist, CT technologist, patient coordinator, and call center) | **Facilitators**  ·Uninsured  **Barriers**  ·Older age  ·Asian/Asian British and mixed ethnicity (compared with White)  ·Unaware of LDCT (inclusion criteria; benefit vs. cost)  ·Fear (false-positive results)  ·Competing priorities  ·Fatalism  ·Blame and stigma | - |
| Huang et al^36^  (2025, China) |  | **Facilitators**  ·Public media  **Barriers**  ·Non-standardized management of health records | **Facilitators**  ·Healthcare logistics  **Barriers**  ·High operational costs  ·Insufficient human resources (doctors, nurses, radiologists, community healthcare providers, and community volunteers) |  | - |
